# Temporal changes of genes associated with intestinal homeostasis in broiler chickens following a single infection with *Eimeria acervulina*

**DOI:** 10.1016/j.psj.2023.102537

**Published:** 2023-01-26

**Authors:** Sara E. Cloft, Katarzyna B. Miska, Mark Jenkins, Monika Proszkowiec-Weglarz, Stanislaw Kahl, Eric A. Wong

**Affiliations:** ⁎ School of Animal Sciences, Virginia Tech, Blacksburg, VA 24061, USA; † Animal Biosciences and Biotechnology Laboratory, Henry A. Wallace Beltsville Agricultural Research Center, Beltsville, MD 20705, USA

**Keywords:** *Eimeria acervulina* surface antigen, crypt depth, host defense peptides, intestinal homeostasis, goblet cells

## Abstract

Infection with the protozoan parasite *Eimeria* can cause the economically devastating disease coccidiosis, which is characterized by gross tissue damage and inflammation resulting in blunted villi and altered intestinal homeostasis. Male broiler chickens at 21 d of age were given a single challenge with *Eimeria acervulina.* Temporal changes in intestinal morphology and gene expression were investigated at 0, 3, 5, 7, 10, and 14 d postinfection (**dpi**). There were increased crypt depths for chickens infected with *E. acervulina* starting at 3 dpi and continuing to 14 dpi. At 5 and 7 dpi, infected chickens had decreased Mucin2 (**Muc2**), and Avian beta defensin (**AvBD**) 6 mRNA at 5 and 7 dpi and decreased AvBD10 mRNA at 7 dpi compared to uninfected chickens. Liver-enriched antimicrobial peptide 2 (**LEAP2**) mRNA was decreased at 3, 5, 7, and 14 dpi compared to uninfected chickens. After 7 dpi, there was increased Collagen 3a1 and Notch 1 mRNA compared to uninfected chickens. Marker of proliferation Ki67 mRNA was increased in infected chickens from 3 to 10 dpi. In addition, the presence of *E. acervulina* was visualized by in situ hybridization (**ISH**) with an *E. acervulina* sporozoite surface antigen (**Ea-SAG**) probe. In *E. acervulina* infected chickens, Ea-SAG mRNA was only detectable on 5 and 7 dpi by both ISH and qPCR. To further investigate the site of *E. acervulina* infection, Ea-SAG and Muc2 probes were examined on serial sections. The Muc2 ISH signal was decreased in regions where the Ea-SAG ISH signal was present, suggesting that the decrease in Muc2 by qPCR may be caused by the loss of Muc2 in the localized regions where the *E. acervulina* had invaded the tissue. *Eimeria acervulina* appears to manipulate host cells by decreasing their defensive capabilities and thereby allows the infection to propagate freely. Following infection, the intestinal cells upregulate genes that may support regeneration of damaged intestinal tissue.

## INTRODUCTION

One of the greatest health and management challenges faced in the poultry industry is the parasitical disease coccidiosis, caused by *Eimeria* spp. which invades epithelial cells along the villi. *Eimeria acervulina* is one of the most common species that infect chickens, preferentially invading the duodenum (Conway and Mckenzie, 2007). Inside infected epithelial cells, *Eimeria* sporozoites multiply asexually increasing in number until the epithelial cell ruptures releasing parasites. The released first generation merozoites invade neighboring epithelial cells and undergo further asexual multiplication (Warren and Ball, 1967). Eventually, sexual reproduction is initiated and fertilized oocysts that emerge from epithelial cells are shed in the feces. These oocysts sporulate and become infectious in the litter environment, leading to a new infection cycle in previously noninfected or nonimmune chickens. The infection-multiplication cycle culminates in widespread tissue damage impairing the functionality of the intestine and reducing the performance of the bird.

In general, few studies have tracked molecular changes following *E. acervulina* infection over 2 wk postinfection, most studies have limited sampling to peak infection, around 7 d postinfection (**dpi**). Previous studies have reported changes in host defense peptides, such as avian beta-defensins (**AvBD**) and liver-enriched antimicrobial peptide 2 (**LEAP2**) (Paris and Wong, 2013; Su et al., 2017) and in intestinal functioning via digestive enzymes and nutrient transporters (Su et al., 2014; Miska and Fetterer, 2017). These studies have shown that in response to *E. acervulina* invasion, LEAP2 mRNA is downregulated as well as brush border nutrient transporters are downregulated, which may reduce the intake of glutamate and other critical amino acids.

To date no research has investigated the change in intestinal homeostasis following *Eimeria* infection. Intestinal homeostasis is the ideal state where the intestine balances nutrient digestion and absorption with maintaining defensive barrier functions without prioritizing one function to the point that it impairs the other. *Eimeria* infection disrupts intestinal homeostasis by invading absorptive enterocytes and redirecting absorbed nutrients as well as causing tissue damage that activates the immune system. Fernando and McCraw (1973) have previously shown that intestinal crypt depth (**CD**) increases and villus height (**VH**) decreases following infection, but no work has investigated the gene expression pattern supporting this change. The stem cell marker gene Olfactomedin 4 can be utilized to evaluate CD as it clearly delineates stem cells in the crypt from cells of the villi (Zhang and Wong, 2018; Liu et al., 2020). Increases in CD likely reflect increases in cell proliferation, though no one has evaluated changes in expression of proliferation genes, such as marker of proliferation Ki67.

Following peak infection of *Eimeria*, VH rebounds likely due to increased replacement cells from the crypt. In a feed additive study, Brautigan et al. (2017) correlated increased VH with increased collagen mRNA and collagen deposition. No research, however, has assessed collagen gene expression following intestinal tissue destruction. Similarly, villi growth would likely be accompanied by increased expression of cell differentiation signaling genes such as Notch 1, which is associated with differentiation into absorptive enterocytes (Fre et al., 2005).

Identification of *Eimeria* within intestinal cells has relied upon visualization of hematoxylin and eosin stained sections, using the unique morphological structures of the parasite to differentiate it from host cell structures (Conway and Mckenzie, 2007). Detection of specific *Eimeria* species relied on the location of lesions but in the past decade molecular advances have allowed the creation of species-specific primers for PCR-based detection methods (Carvalho et al., 2011). Past attempts at in situ hybridization (**ISH**) for *Eimeria* have targeted viruses infecting the parasite (Lee and Fernando, 1998; Del Cacho et al., 2001).

The main objective of this study was to characterize the change in intestinal homeostasis over time following a single challenge with *E. acervulina* in naïve broiler chickens by examining gene expression and mucosal morphology. Additionally, ISH was utilized to localize *E. acervulina* in duodenal cells of infected broilers.

## MATERIALS AND METHODS

### Animals and Tissue Sampling

All studies were carried out under ARS IACUC protocol #18-025 by the Beltsville Agricultural Research Center (**BARC**) Animal Care and Use Committee and conducted at the Animal Biosciences and Biotechnology Laboratory (USDA Agricultural Research Service, Beltsville, MD). Two hundred eighty-eight male Ross 708 broilers (1 d of age) were obtained from a local hatchery (Longnecker's Hatchery, Elizabethtown, PA) and placed into 1 m^2^ wire pens (25 chicks per pen). At 19 d of age, chickens were placed in wire battery cages (Petersime finisher units, Petersime, Gettysburg, OH), with 4 chickens per cage. Chickens were fed a corn-soybean meal diet that met the nutritional requirements as described by the National Research Council (NRC, 1994). At 21 d of age, chickens were orally gavaged with 2 mL of either sterile water (uninfected) or with 100,000 *E. acervulina* oocysts from USDA BARC stock (infected). After infection, chickens were returned to cages (36 cages per treatment, 4 chickens per cage) on opposite sides of the room to prevent cross-contamination.

Approximately 3 h after infection, 0 dpi samples were collected from both infected and uninfected groups. Samples were also collected at 3, 5, 7, 10, and 14 d postinfection (**dpi**) from 6 different cages at each timepoint. At each sampling point all 4 chickens/cage and feed were weighed to determine body weight gain (**BWG**), feed intake (**FI**), and feed conversion ratio (**FCR**). Two chickens of average weight were selected from each of 6 cages and euthanized by cervical dislocation for intestinal sampling. One of the 2 chickens was used for gene expression analysis (n = 6), while the other chicken was used for histological analysis (n = 6). Following euthanasia, blood samples were collected by cardiac puncture and placed into EDTA containing tubes. Samples were centrifuged at 2,000 × *g* at 4°C to collect plasma for carotenoid measurements using previously described methods (Allen, 1987). Carotenoid levels along with decrease in BWG were used to verify a successful *E. acervulina* infection. An intact 5 cm segment from the center of the duodenal loop was dissected and rinsed with sterile PBS and placed into neutral buffered formalin for later histological analysis. Twenty-four hours after collection, samples were transferred to 70% ethanol and kept until embedding in paraffin. l Duodenal segments from a replicate chicken were diced, snap frozen in liquid nitrogen, stored at −80°C and used for qPCR analysis.

### Identification of Stem Cells and Morphological Measurements

The segments collected for histological analysis were embedded in paraffin (StageBio, Mount Jackson, VA). Formalin-fixed, paraffin embedded tissues were sectioned (5–6 µm) using a microtome and mounted on Superfrost-Plus glass slides (Electron Microscopy Sciences, Hatfield, PA). In situ hybridization was conducted using the RNAscope (Advanced Cell Diagnostics, Newark, CA) method with a singleplex olfactomedin 4 (Olfm4) probe (NM_001040463.1) and the RNAscope 2.5 HD Assay–BROWN detection kit (Advanced Cell Diagnostics). Slides were counterstained with 50% Gill's hematoxylin no. 1 (Sigma-Aldrich, St. Louis, MO), rinsed in distilled water, and placed in 0.02% ammonia water to turn the purple stain to blue. Slides were sealed with VectaMount (Vector Lab, Burlingame, CA) and a glass coverslip.

Brightfield microscopy images were captured using a Nikon Eclipse 80i microscope with a DS-Ri1 digital camera (Nikon Instruments, Inc., Melville, NY). Villus height and CD were measured on 40× magnification images using Image J software from the National Institutes of Health (Bethesda, MD). Olfm4 is a marker of intestinal stem cells and was used to define the functional crypt for CD measurements on ISH images (Zhang and Wong, 2018). For hematoxylin stained images, CD was determined based on morphological differences between the crypt and villi structures. VH was measured from the top of the functional crypt to the tip of the villus. Approximately 75 CD measurements and 30 VH measurements were taken per sample (n = 6 chickens per treatment/timepoint).

### RNA Extraction and Quantitative PCR

Snap frozen samples were homogenized with TriReagent using a tissue lyser and total RNA was extracted using the Directzol RNA mini prep columns (Zymo Research, Irvine, CA). RNA concentration and purity were determined using a Nanodrop 1000 spectrophotometer (Thermo Fisher Scientific, Waltham, MA). Complementary DNA was synthesized using 1 μg of total RNA and the Applied Biosystems high capacity cDNA reverse transcription kit (Thermo-Fisher Scientific). Gene expression was determined by quantitative PCR (**qPCR**). The qPCR reactions consisted of 5 µL Fast SYBR Green Master Mix (Thermo Fisher Scientific), 1 µL of forward primer (5 µM), 1 µL of reverse primer (5 µM), and 1.5 µL of cDNA (diluted 1:30). Duplicate qPCR reactions were performed using an Applied Biosystems 7500 Fast Real-time PCR system (Thermo Fisher Scientific) using the default fast program (95°C for 20 s, 40 cycles of 90°C for 3 s and 60°C for 30 s). Primers were designed using Primer Express 3.0 (Thermo Fisher Scientific) and are listed in Table 1. The *E. acervulina* major sporozoite surface antigen gene (**Ea-SAG**) was identified as a potential parasite marker because of its conserved domain (in the superfamily cl12617 Sporozoite TA4 surface antigen pfam11054) shared between *E. acervulina, E. maxima,* and *E. tenella*. There was no similarity to the chicken genome based on a BLASTn search (Boratyn et al., 2013). Chicken ribosomal protein L4 (**RPL4**) and ribosomal protein lateral stalk subunit P0 (**RPLP0**) were used as reference genes. The geometric mean of the Ct value for RPLP0 and RPL4 was subtracted from the Ct value for the target gene to obtain the ΔCt value for each sample. The average ΔCt value of the uninfected chickens at d0 was used as the calibrator to calculate ΔΔCt and fold change using the 2^−ΔΔCt^ method (Schmittgen and Livak, 2008).

**Table 1:** Primers for quantitative PCR.

Gene name	Forward/Reverse Primers (5’ to 3’)	Amplicon Size (bp)	Accession no.^1^
Avian β-defensin 1 (AvBD1)	GAGTGGCTTCTGTGCATTTCTG/ TTGAGCATTTCCCACTGATGAG	62	NM_204993.1
Avian β-defensin 6 (AvBD6)	GCCCTACTTTTCCAGCCCTATT/ GGCCCAGGAATGCAGACA	63	NM_001001193.1
Avian β-defensin 10 (AvBD10)	CAGACCCACTTTTCCCTGACA/ CCCAGCACGGCAGAAATT	64	NM_001001609.2
Liver-enriched antimicrobial peptide 2 (LEAP2)	CTCAGCCAGGTGTACTGTGCTT/ CGTCATCCGCTTCAGTCTCA	66	NM_001001606.1
Mucin 2 (Muc2)	CTGATTGTCACTCACGCCTTAATC/ GCCGGCCACCTGCAT	147	JX284122.1
Olfactomedin 4 (Olfm4)	TTGCCGGATACCACCTTTCC/ TTTCTGCAAGAGCGTTGTGG	72	NM_001040463.1
Marker of proliferation mKi67 (Ki67)	CACAGGCAAAGGCTGTCAAA/ TCCGTGCAATTTTCCTTGCT	63	XM_015289038.4
Notch homolog-1 (Notch1)	GAGGATCCATCGTCTACTTGGAA/ ATCGGTTGCGCTCTGGAA	81	NM_001030295.1
Collagen 3a1 (Col3a1)	GGCATTCCTCCGCATCCT/ TTGCAGTGGTAGGTGATGTTCTG	57	NM_205380.2
*Eimeria acervulina* sporozoite surface antigen (Ea-SAG)	TGAGTTCCGCACGCAAGA/ TCGATGTCTCGGCAACGAA	56	XM_013394133.1
Ribosomal protein large subunit P0 (RPLP0)	GCGATTGCTCCCTGTGATG/ TCTCAGGTCCGAGACCAGTGT	58	NM_204987.2
Ribosomal protein large subunit 4 (RPL4)	TCAAGGCGCCCATTCG/TGCGCAGGTTGGTGTGAA	63	NM_001007479.1

1 Primer sequences were designed for regions of consensus between all published variants.

### Localizing Eimeria Acervulina in the Duodenum

In situ hybridization was conducted on samples collected for histological analysis using the RNAscope methodology with a singleplex Ea-SAG (XM_013394133.1) probe and the RNAscope 2.5 HD Assay–BROWN detection kit (n = 3 to 6). A second set of slides was prepared as serial sections and ISH was conducted with the Ea-SAG and Muc2 (JX284122.1) singleplex probes on serial sections and detected using the RNAscope 2.5 HD Assay–BROWN detection kit. These slides were counterstained with first Alcian Blue (MilliporeSigma, St. Louis, MO) to detect acidic mucus glycoproteins and then 50% Gill's hematoxylin no. 1 (Sigma-Aldrich). Slides were sealed with VectaMount and a glass coverslip. Brightfield microscopy images were captured using a Nikon Eclipse E600 microscope with a DS-Fi1 digital camera.

### Statistical Analysis

Performance data (BW, BWG, FI, FCR) and plasma carotenoid concentration were analyzed by 2 factor ANOVA considering time postinfection and infection status via JMP v15.0 (SAS Institute Cary, NC). Gene expression data were transformed logarithmically to conform to normality for statistical analysis and are presented as back-transformed means (Jørgensen and Pedersen, 1998). Data were analyzed for the effect of time by infection status using a one-way ANOVA of the data subset by infection status via JMP v15.0. When significant ANOVA results were observed, Tukey's Honestly Significant Difference test was conducted for mean separation. Additionally, data were analyzed for the effect of infection status at each timepoint by a student's *t* test. Morphological data were unable to be transformed to meet conditions of normal distribution, due to unequal variances; therefore, data were analyzed using the nonparametric Welch's one-way test for the effect of infection status at each timepoint. Statistical significance was established at *P* ≤ 0.05 for all analyses.

## RESULTS

### Growth Performance Measurements

There was no significant interaction between infection status and dpi for any growth performance measurements, therefore, only main effects (interaction and dpi) are discussed below for BW, BWG, FI, and FCR (Table 2). All growth performance measurements were different over dpi. Chickens at 14 dpi had the greatest BW, BWG, and FI compared to all earlier dpi. FCR was significantly greater at 14 dpi in comparison to 3 and 5 dpi but not different from 7 and 10 dpi. BWG of infected chickens was 87 g less than uninfected chickens during the study period. Additionally, BW and FI tended (*P* < 0.1) to be lower in infected chickens than uninfected chickens.

**Table 2:** Growth performance data of infected and uninfected broiler chickens.^1^

Day postinfection × Infection Status		Body weight (kg)	Body weight gain^2^ (kg)	Feed intake (kg)	Feed conversion ratio
3 dpi	Uninfected	1.05	0.27	0.30	1.11
	Infected	1.03	0.21	0.20	0.96
5 dpi	Uninfected	1.21	0.44	0.42	1.02
	Infected	1.20	0.36	0.40	1.15
7 dpi	Uninfected	1.36	0.55	0.66	1.19
	Infected	1.30	0.49	0.61	1.25
10 dpi	Uninfected	1.68	0.85	1.14	1.34
	Infected	1.58	0.76	1.05	1.38
14 dpi	Uninfected	1.99	1.16	1.64	1.43
	Infected	1.85	1.02	1.65	1.83
SEM^3^		0.06	0.05	0.04	0.15
Main effect Day postinfection					
3 dpi		1.04^d^	0.24^d^	0.25^e^	1.04^b^
5 dpi		1.20^cd^	0.40^c^	0.41^d^	1.08^b^
7 dpi		1.33^c^	0.52^c^	0.63^c^	1.22^ab^
10 dpi		1.63^b^	0.81^b^	1.09^b^	1.36^ab^
14 dpi		1.92^a^	1.09^a^	1.64^a^	1.63^a^
SEM^3^		0.04	0.04	0.03	0.11
Main effect infection status					
Uninfected		1.46	0.65^a^	0.83	1.22
Infected		1.39	0.57^b^	0.78	1.31
SEM^3^		0.03	0.02	0.02	0.07
Analysis of variance			Probabilities^4^		
Day postinfection × infection status		0.79	0.91	0.67	0.47
Day postinfection		*<0.0001*	*<0.0001*	*<0.0001*	*0.0015*
infection status		0.073	*0.0085*	0.067	0.31

1 Each value represents the least-square means from 6 replicate cages with 4 male chickens/cage. Chickens at 21 d of age were infected with 100,000 *Eimeria acervulina* oocysts (Infected) or sham infected with sterile water (Uninfected) and evaluated during 14 d postinfection (dpi).

2 Body weight gain is calculated since 0 dpi. On 0 dpi average body weight = 0.85 kg.

3 SEM = pooled standard error.

4 Italicized *p*-values are statistically significant (*P*< 0.05).

a,b,c,d,e Means in the same column with different superscripts are significantly different (*P* < 0.05) based on Tukey HSD mean separation.

**Figure 1:**
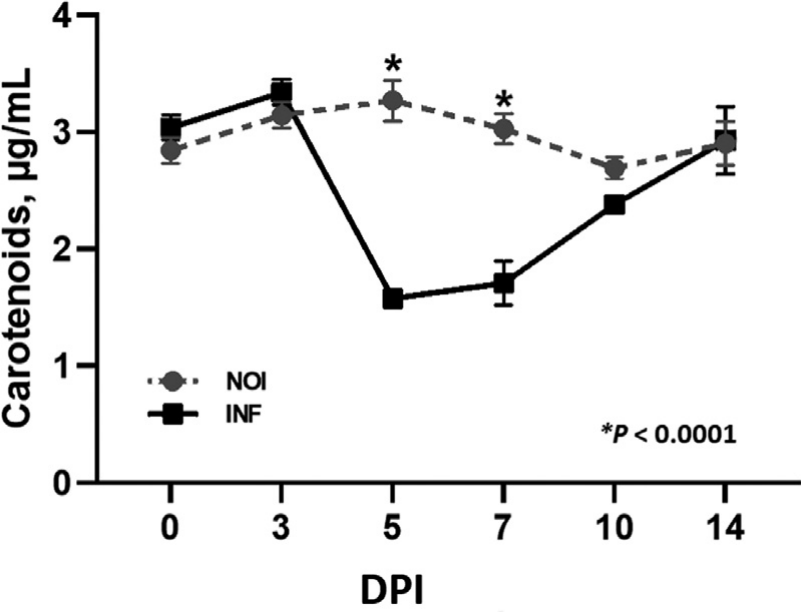
Plasma carotenoid concentrations in uninfected and *Eimeria acervulina* infected chickens. Chickens were infected at 21 d of age with either 100,000 *E. acervulina* oocysts (Infected) or sham infected with sterile water (Uninfected) and sampled at 0, 3, 5, 7, 10, and 14 d postinfection (dpi). All values are means ± SEM. Asterisks (*) indicate value significantly different (*P* < 0.0001) from the corresponding uninfected timepoint.

Plasma carotenoid concentrations in infected and uninfected chickens were evaluated throughout the study (Figure 1) as an indirect means of assessing *E. acervulina* infection as described in Allen and Fetterer (2000). Infected broilers had significantly reduced plasma carotenoids on 5 and 7 dpi compared to uninfected chickens. The significant plasma carotenoid and BW decreases in infected birds indicates an observable *E. acervulina* infection.

### Morphology Measurements

**Figure 2:**
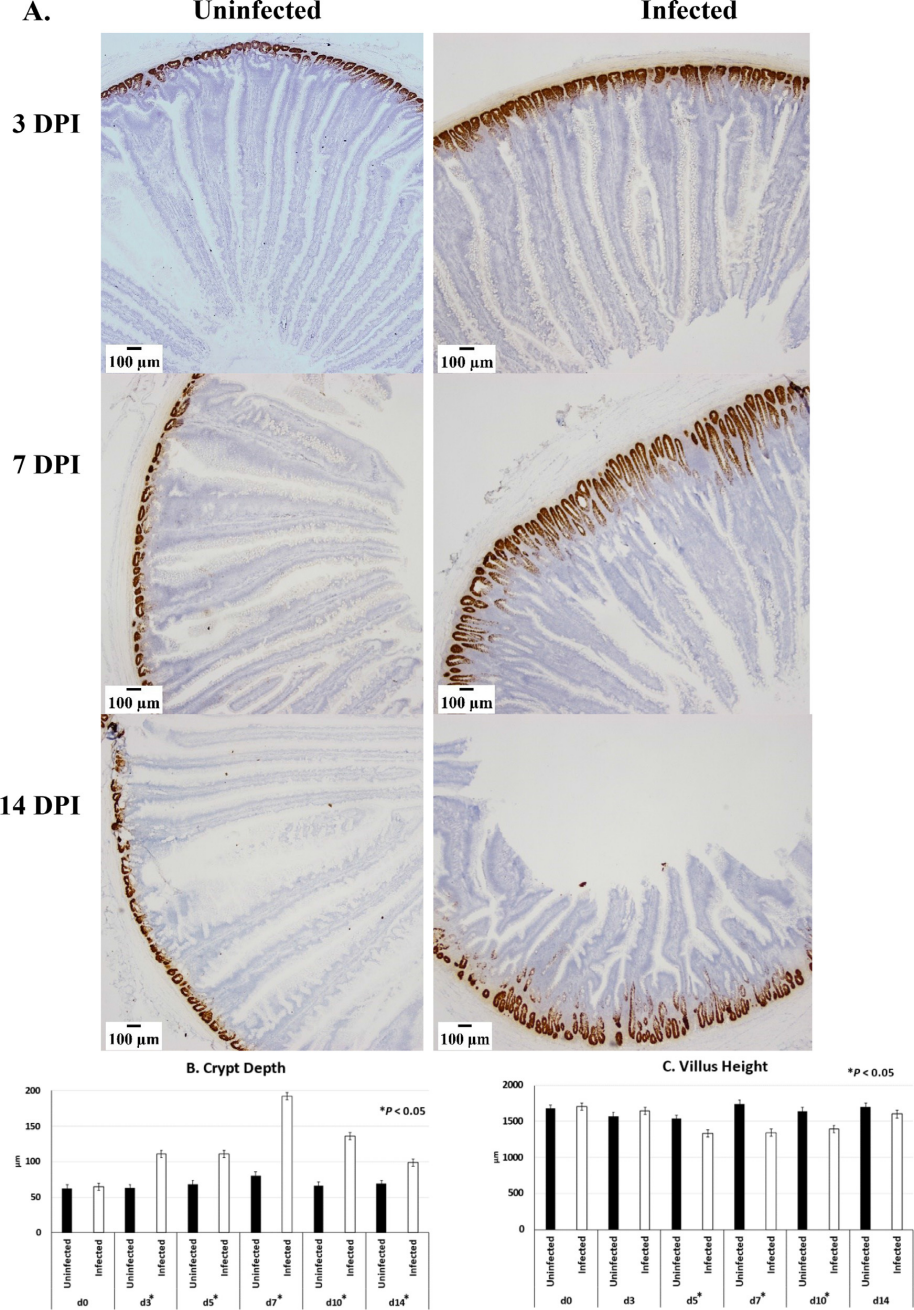
Crypt elongation following infection with *Eimeria acervulina*. (A) Expression of Olfm4 mRNA by in situ hybridization in the duodenum of broiler chickens that were infected at 21 d of age with either 100,000 *E. acervulina* oocysts (Infected) or sham infected with sterile water (Uninfected) and sampled at 0, 3, 5, 7, 10, and 14 d postinfection (dpi). All tissues were counterstained with 50% hematoxylin. Images were captured at 40X magnification (n = 6). (B) Crypt Depth (CD) and (C) villus height (VH) were measured on duodenal sections stained for Olfm4 by in situ hybridization. Measures were analyzed by infection status using the nonparametric Welch's one-way test. Significances (*P* < 0.05) are indicated by asterisks (*) along the x-axis.

Olfactomedin 4 is a stem cell marker and thus can be used to mark the crypt. The ISH for Olfm4 mRNA revealed clear differences in CD between infected and uninfected groups starting at 3 dpi and continuing through 14 dpi (Figures 2A and 2B). To compare the measurement of CD as determined with Olfm4 by ISH with the CD by morphology with hematoxylin staining, a set of serial sections were compared (Table 3). The CD difference between infected and uninfected groups was observed again, with the infected group having larger CD measures than uninfected chickens. In comparing the CD measures with the different methods, there was a difference between hematoxylin stained and Olfm4 ISH stained CD measures, with hematoxylin measures greater than the Olfm4 measures. As expected, VH was decreased for infected chickens during the peak of infection on 5 and 7 dpi as well as 10 dpi compared to uninfected chickens (Figure 2C). By 14 dpi the VH of infected chickens was the same as uninfected chickens.

**Table 3:** Comparison of crypt depth measures between Olfm4-stained crypts and hematoxylin stained crypts.^1^

Day postinfection	Stain	Uninfected (μm)	Infected (μm)
0	Olfm4	95.8	70.8
	Hematoxylin	121.4	91.4
3	Olfm4	78.6	146.4
	Hematoxylin	103.8	168.0
5	Olfm4	86.0	172.5
	Hematoxylin	101.1	231.8
7	Olfm4	130.6	223.7
	Hematoxylin	145.3	256.6
10	Olfm4	81.5	146.0
	Hematoxylin	109.8	182.6
14	Olfm4	77.1	128.7
	Hematoxylin	105.2	129.5
SEM^2^		69.3	52.4
Welch's unequal variance test		*Probabilities*	
Stain		0.048	
Infection status		< 0.0001	
Day postinfection		0.0027	

1 Measures following Olfm4 in situ hybridization using RNAscope 2.5 HD kit (Brown) or Hematoxylin staining in broiler chickens that were infected at 21 d of age with 100,000 *Eimeria acervulina* oocysts (Infected) or sham infected with sterile water (Uninfected) and sampled at 0 (3 h postinfection), 3, 5, 7, 10, and 14 d post infection (dpi). Measurements were taken at 40 X magnification (n = 6) using ImageJ.

2 SEM = pooled standard error.

### Intestinal Gene Expression

**Figure 3:**
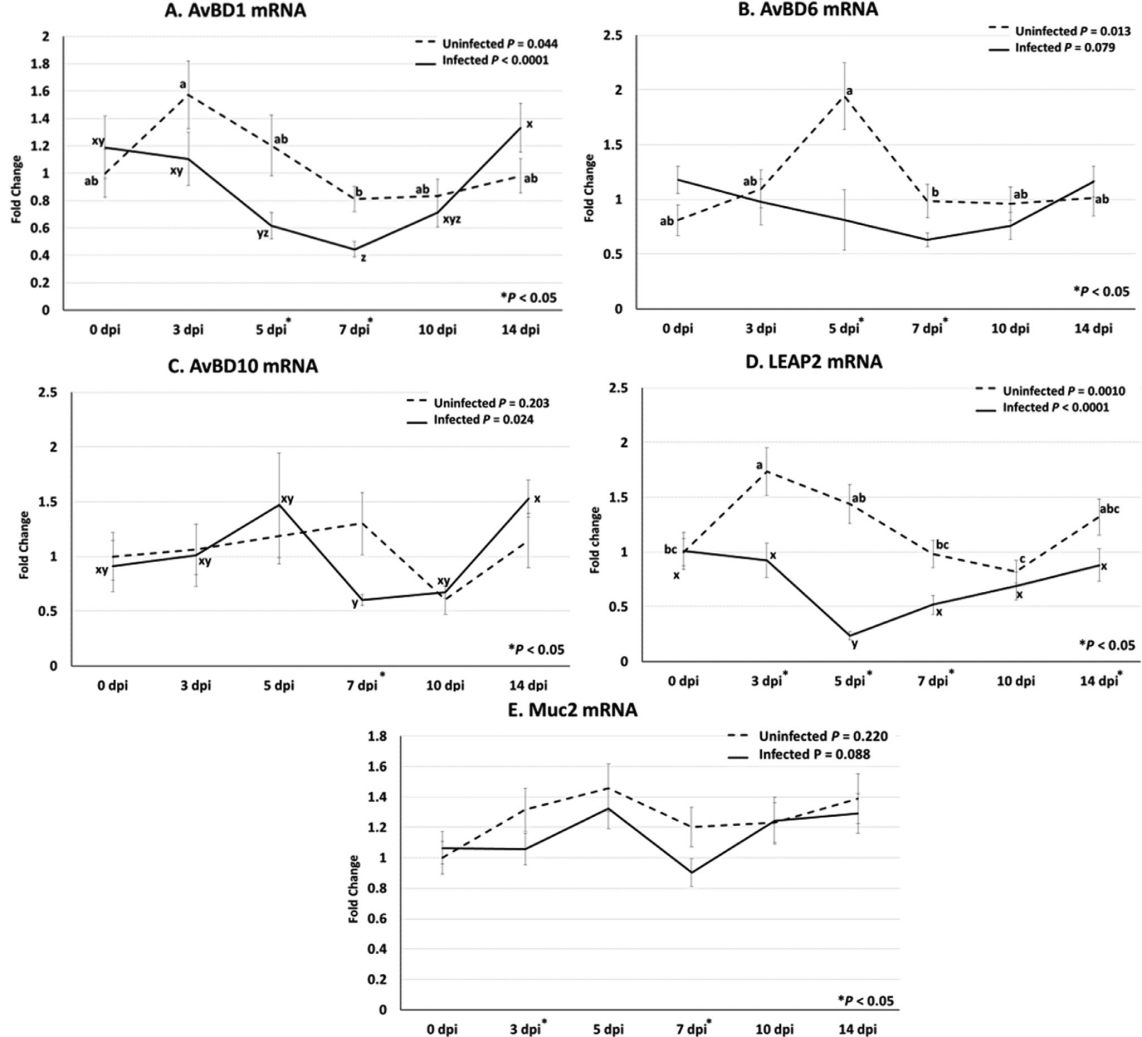
Avian beta defensins (AvBD), Liver-enriched antimicrobial peptide 2 (LEAP2), and Mucin 2 (Muc2) mRNA profiles following infection with *Eimeria acervulina.* Chickens were infected at 21 d of age with either 100,000 *E. acervulina* oocysts (Infected) or sham infected with sterile water (Uninfected) and sampled at 0, 3, 5, 7, 10, and 14 d postinfection (dpi). Duodenal tissues were analyzed by relative qPCR (n = 6) for expression of AvBD1 (A), AvBD6 (B), AvBD10 (C), LEAP2 (D), and Muc2 (E) mRNA. Values presented are the means ± standard error. A one-way ANOVA was used to analyze Infected and Uninfected data separately and when significant, Tukey HSD was used for mean separation. Significant mean separations are indicated by “abc” for Uninfected means and “xyz” for Infected means. Additionally, a *t* test was conducted for each dpi, independent of the ANOVA, to assess the effect of infection. Significant differences (*P* < 0.05) are indicated by asterisks (*) along the x-axis.

Avian beta-defensins, LEAP2, and Muc2 are part of the defensive barrier function of the intestine protecting the body from pathogens. Infected chickens had decreased AvBD1 and AvBD6 mRNA compared to uninfected chickens at 5 and 7 dpi (Figures 3A and 3B). Though on 5 dpi, the difference in AvBD6 mRNA is more likely due to an unexplained increase in uninfected chickens rather than the decrease in infected chickens. AvBD10 mRNA was also decreased in infected chickens but only on 7 dpi (Figure 3C). Additionally, LEAP2 mRNA was decreased in infected chickens on 3, 5, 7, and 14 dpi between infected and uninfected groups (Figure 3D). Muc2 mRNA was decreased in infected chickens compared to uninfected chickens at 3 and 7 dpi (Figure 3E).

**Figure 4:**
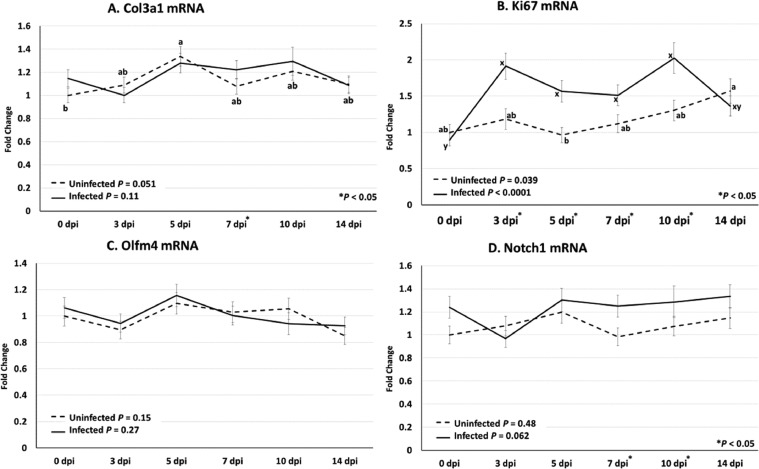
Collagen 3a1 (Col3a1), Marker of proliferation Ki67, Olfactomedin 4 (Olfm4), and Notch 1 mRNA profiles following infection with *Eimeria acervulina.* Chickens were infected at 21 d of age with either 100,000 *E. acervulina* oocysts (Infected) or sham infected with sterile water (Uninfected) and sampled at 0, 3, 5, 7, 10, and 14 d postinfection (dpi). Duodenal tissues were analyzed by relative qPCR (n = 6) for expression of Col3a1 (A), Ki67 (B), Olfm4 (C), and Notch 1 (D) mRNA. Values presented are the means ± standard error. A one-way ANOVA was used to analyze Infected and Uninfected data separately and when significant Tukey HSD was used for mean separation. Significant mean separations are indicated by “ab” for Uninfected means and “xy” for Infected means. Additionally, a *t* test was conducted for each dpi, independent of the ANOVA, to assess the effect of infection. Significant differences (*P* < 0.05) are indicated by asterisks (*) along the x-axis.

Col3a1, Ki67, Olfm4, and Notch 1 are genes associated with maintaining intestinal functioning and homeostasis. Collagen 3a1 mRNA was increased in infected chickens compared to uninfected chickens on 7 dpi (Figure 4A). While the difference was statistically significant, the increase was small. The proliferative cell marker Ki67 mRNA was greater in infected chickens compared to uninfected chickens at 3, 5, 7, and 10 dpi (Figure 4B). The stem cell marker gene, Olfm4 mRNA was not different between infected and uninfected chickens at any timepoint (Figure 4C). Notch 1 mRNA was increased in infected chickens at 7 and 10 dpi compared with uninfected chickens (Figure 4D).

Many of the genes analyzed showed temporal changes in mRNA abundance. AvBD1 (Figure 3C) and LEAP2 (Figure 3D) mRNA showed decreased expression at 7 and 5 dpi, respectively, compared to 0 and 14 dpi in infected chickens, which corresponded to the time around peak infection. In contrast Ki67 mRNA (Figure 4B) showed an increase from 0 to 3 dpi, which remained elevated to 10 dpi. For uninfected chickens, there was some unexpected variability in the temporal expression of AvBD1, AvBD6, LEAP2, Col3a1, and Ki67 mRNA.

### Localizing *Eimeria acervulina* In Situ

*E. acervulina* sporozoite surface antigen (**Ea-SAG**) mRNA was only detectable by qPCR in infected chickens on 5 and 7 dpi (Ct value < 30) and not detectable in any uninfected chickens at any timepoint. All 6 infected chickens had an average Ct of 19 on 5 dpi and average Ct of 26 on 7 dpi. Due to the absence of detection of Ea-SAG in any uninfected chickens and during d 0, 3, 10, and 14 dpi in infected chickens, no quantitative statistics were used to analyze the difference.

**Figure 5:**
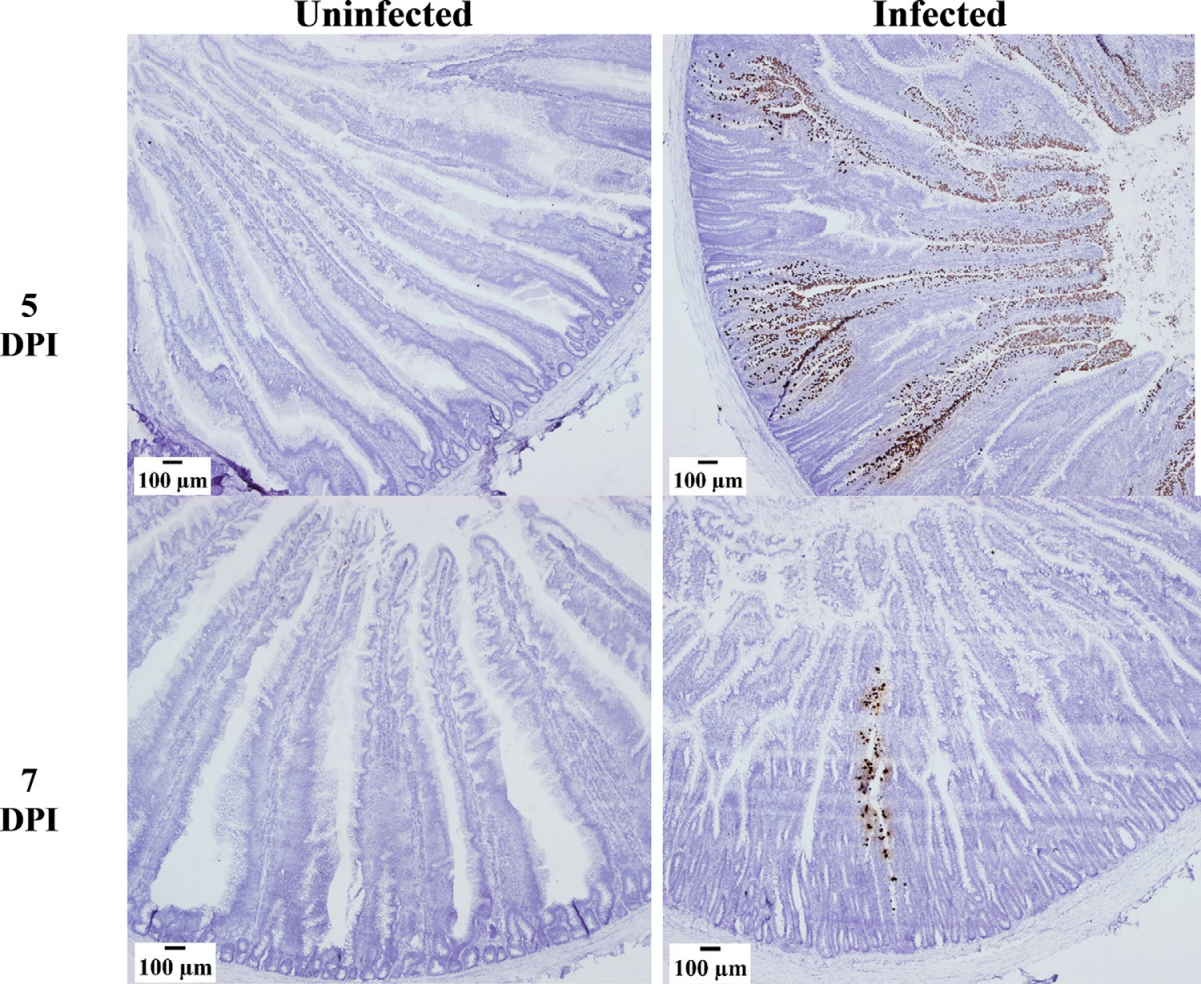
In situ hybridization analysis of *Eimeria acervulina* sporozoite surface antigen (Ea-SAG) mRNA following infection with *E. acervulina.* Chickens were infected at 21 d of age with either 100,000 *E. acervulina* oocysts (Infected) or sham infected with sterile water (Uninfected). Cells in the duodenum at 5 and 7 d postinfection (dpi) expressing Ea-SAG mRNA were detected using the RNAscope 2.5 HD kit (Brown) in situ hybridization method. All tissues were counterstained with 50% hematoxylin. Images were captured at 40X magnification (n = 6).

In situ hybridization with an Ea-SAG probe was conducted for all timepoints and treatments in this study. Presence of Ea-SAG mRNA was observed only in infected chickens on 5 and 7 dpi (Figure 5), confirming the qPCR results. At 5 dpi, Ea-SAG expression in situ was present along the villi and in the crypt. By 7 dpi the Ea-SAG signals were restricted to a few contained regions along the villi.

**Figure 6:**
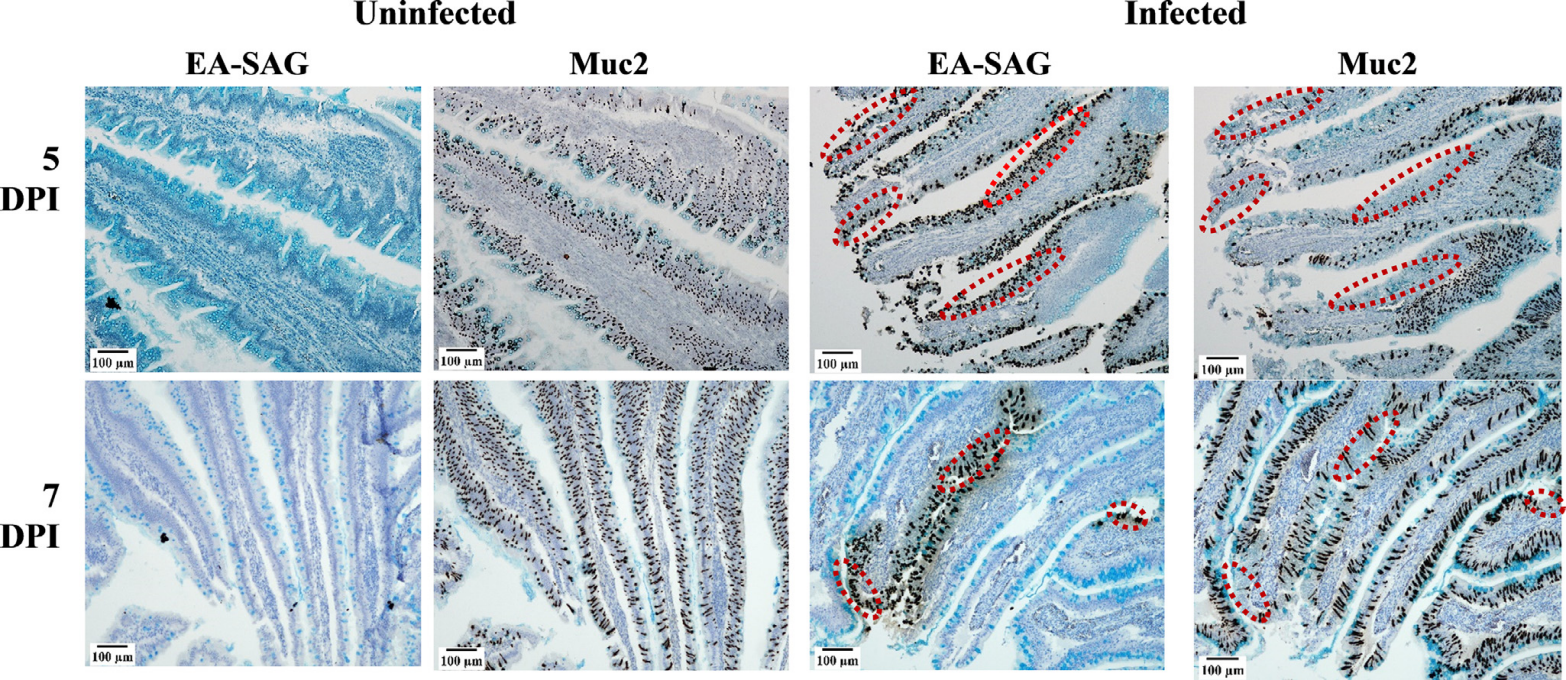
*In situ* hybridization analysis of *Eimeria acervulina* sporozoite surface antigen (Ea-SAG) and Mucin 2 (Muc2) mRNA following infection with *E. acervulina*. Chickens were infected at 21 d of age with either 100,000 *E. acervulina* oocysts (Infected) or sham infected with sterile water (Uninfected). Serial sections of the duodenum of chickens at 5 and 7 d postinfection (dpi) were analyzed by in situ hybridization using RNAscope 2.5 HD kit (Brown). Cells expressing Ea-SAG mRNA or Muc2 mRNA are stained brown. All tissues were counterstained with alcian blue to detect the mucin glycoprotein (cells appearing blue on all images) and 50% hematoxylin. Images were captured at 100X magnification (n = 3). Red dashed ovals on infected images indicate areas of decreased Muc2 mRNA expression in the same regions where the Ea-SAG mRNA signal is present.

To better understand the site of *E. acervulina* invasion in the duodenum, the location of Ea-SAG mRNA and Muc2 mRNA were analyzed on serial sections. The Muc2 mRNA signal was reduced in the locations where the Ea-SAG mRNA signal was present (Figure 6; marked by red dashed ovals).

## DISCUSSION

### Defensive Barrier Functions Suppressed Following Infection

Previous studies have shown downregulation of defensive barrier function and specifically LEAP2 mRNA as a consequence of *Eimeria* sp*.* infection (Paris and Wong, 2013; Su et al., 2014, 2017). Paris and Wong (2013) hypothesized that *Eimeria* sp*.* invade epithelial cells and downregulate LEAP2 to prevent their own destruction. In response, the invaded cell downregulates amino acid transporters lowering resources available to the parasite. Essentially, once the *Eimeria* sp. blocks the defensive capabilities of the epithelial cell, the cell may attempt to starve and destroy the invading parasite as a way to stop the infection. Previously, researchers have showed that *Eimeria* spp*.* are capable of manipulating a variety of host functions such as inhibiting host cell apoptosis through Nuclear Factor κβ activation (del Cacho et al., 2004; Lutz et al., 2011); although, no mechanism of downregulation for LEAP2 has been defined.

Along with LEAP2, AvBD have been reported to be downregulated on 7 dpi perhaps by the same mechanism (Su et al., 2017). It is worth noting that timepoints other than 7 dpi have not been investigated in many studies so there are few studies that allow a direct comparison. It is likely that the downregulation of defense barrier functions causes a loss in protective function of the intestine, which explains the intensity of symptoms (decreased FI, slowed BWG, and decreased plasma carotenoids) displayed by infected chickens around peak infection. Our results agree with this hypothesis as infected chickens had decreased LEAP2 and AvBD1 mRNA on 5 or 7 dpi, which coincided with the lowest BWG and FI in infected chickens.

The peak of infection is reached between 5 and 7 dpi in solo challenge studies because the lifecycle of *E. acervulina* is fixed so that after each sporozoite undergoes 2 to 3 rounds of asexual reproduction it transitions to sexual reproduction (Warren and Ball, 1967). If there is no re-infection then the parasite will exhaust its reproductive potential around 7 dpi when oocyst shedding typically peaks (Allen and Fetterer, 2002). Following peak infection, the intestine begins to recover. In this study, there was a pattern of increasing AvBD1 and AvBD10 mRNA after 7 dpi, which became more similar to those of uninfected chickens at 10 and 14 dpi. By contrast, LEAP2 mRNA began increasing after 5 dpi but remained less than the uninfected group on 7 and 14 dpi. In addition to being a sign of recovery, the increase in defensins post-peak may be a sign of immunity developing, as it has been well established that chickens develop immunity to *Eimeria* sp. infections following 3 to 4 reinfection cycles (Rose, 1987).

In this study there were multiple instances of changes in mRNA abundance over time in uninfected chickens, for both defense and homeostasis genes. There is no clear explanation for this result as cross-contamination was minimized through housing treatment groups on opposite sides of the same room. Further, uninfected chickens maintained a low FCR and higher FI and BWG compared to the infected group indicating they were not infected. Considering the similar management and performance metrics, and that both defensive and homeostasis genes varied in uninfected chickens, it is likely that the gene expression changes are due to the fact that the intestine is not static and gene expression changes in healthy chickens is part of maintaining intestinal homeostasis.

### Morphological Changes Associated With Intestinal Recovery

Crypt elongation following *Eimeria* sp. infection has been reported previously. Following infection with *E. acervulina,* duodenal crypt elongation was observed from 3 to 12 dpi compared with uninfected chickens (Fernando and McCraw, 1973). In a follow-up study, the elongation was attributed to increased duodenal crypt cell proliferation rate starting at 2 dpi in infected chickens preceding the observed crypt elongation (Fernando and McCraw, 1977). Crypt depth increases are considered to be a result of damage to epithelial cells lining the villi or increased epithelial cell turnover requiring replacement cells from the crypt to prevent loss of absorptive surface area. Crypt elongation associated with regenerating villi can be seen following bacterial diseases in chickens (Cao et al., 2013; Gottardo et al., 2016) and generally following inflammation in the intestine (Sprinz, 1962). The bacterium *Lawsonia intracellularis* caused increased cell proliferation and crypt elongation without increasing intestinal stem cells, likely stimulating precursor cell proliferation outside of the crypt (Huan et al., 2017). Consistent with this idea is the increase in the marker of proliferation Ki67 in the current study. Ki67 mRNA was upregulated from 3 to 10 dpi in infected chickens compared to uninfected chickens.

Olfactomedin 4 expression has been previously shown to be restricted to cells within the intestinal crypt in chickens and thus serves as a robust stem cell marker (Zhang and Wong, 2018). The crypt elongation during *Eimeria* infection may be a reflection of Olfm4 mRNA retention by migrating cells due to the increased proliferation and migration rates up the villi. This has been described in mice following radiation damage, where Olfm4 is thought to play a special role in crypt proliferation after a disruption to intestinal homeostasis (Itzkovitz et al., 2012). Olfm4 mRNA abundance assessed by qPCR did not change over time in the infected group, which appeared contradictory to the ISH results. However, qPCR and ISH do not always match, as qPCR assesses expression at the whole tissue level, while ISH localizes gene expression to specific cells. Thus, the combination of qPCR and ISH provides a more complete analysis of gene expression. Despite the apparent disagreement between qPCR and ISH for Olfm4, the CD as determined by morphology also showed elongation like Olfm4 staining.

Changes in homeostasis gene expression coincide with the villi recovery observed in this study. Following peak-infection, the villi growth rate of infected chickens increased rapidly from 7 to 14 dpi. At 7 dpi, Col3a1 mRNA increased in infected chickens compared to uninfected chickens, potentially supporting the recovery of the villi. Increases in mRNA abundance of multiple collagen genes coinciding with VH increases have been reported previously (Brautigan et al., 2017). Also, the increase in Notch 1 mRNA during 7 and 10 dpi was likely a consequence of that recovery as the villus requires mature enterocytes to replace damaged surface area (Tian et al., 2015). The Notch pathway controls the differentiation of epithelial cells migrating up the villi. Specifically, Notch 1 is expressed within precursor cells committed to the absorptive lineage (Fre et al., 2005). As evidenced by the increased CD, recovering VH and increased Ki67 mRNA, it seems that cell proliferation was increased following *E. acervulina* infection.

Plasma carotenoids are an established indirect indicator of *Eimeria* spp. infection in chickens (Allen, 1987; Allen and Fetterer, 2000; Rochell et al., 2016) as it is a rapid marker of mucosal disruption (Conway et al., 1993). The proposed mechanism for reduced plasma carotenoids is due to impaired fat absorption during *Eimeria* spp. infection, likely due to epithelial cell damage (Adams et al., 1996). The plasma carotenoid values in the current study follow the villi recovery observed. In the infected group, plasma carotenoids were lowest at 5 and 7 dpi when VH was also the lowest. By 14 dpi, both plasma carotenoids and VH were the same in the infected and uninfected groups.

### *Eimeria Acervulina* Infection Corresponds to Changes in Localized Gene Expression

Expression of Ea-SAG mRNA was only detectable by ISH at 5 and 7 dpi, corresponding to the points of greatest infection severity as determined by plasma carotenoids and performance metrics. This sporozoite antigen was expected to be detectable on 3 dpi. It is possible that the mild challenge and low sporozoite numbers associated with the challenge dose, caused an extended time for *Eimeria* to reach a detectable level. Further analysis of the Ea-SAG signal at higher magnifications (images not shown), showed that the Ea-SAG signal detected at 5 and 7 dpi did not mark all of the parasites, as identified by morphology. The Ea-SAG gene is a sporozoite surface antigen so it is possible that only the sporozoite stage of *E. acervulina* produced this mRNA. The Ea-SAG gene was chosen as a potential parasite marker based on the conserved domain in the superfamily cl12617 Sporozoite TA4 surface antigen pfam11054 that was shared between *E. acervulina, E. maxima*, and *E. tenella* species (Brothers et al., 1988). If this is the case, alternative marker genes could be developed to highlight stage-specific progression of infection.

The signal reduction in Muc2 mRNA seen near Ea-SAG ISH signals corresponded to the reported decrease in Muc2 mRNA in infected chickens compared to uninfected chickens. Previous research has reported a decrease in Muc2 mRNA in the intestine of challenged chickens following *E. acervulina* (Wickramasuriya et al., 2021) or mixed *Eimeria* species challenges (Cox et al., 2010; Tan et al., 2014; Chen et al., 2015). Based on the images collected in this study, it may be that the decreased Muc2 mRNA abundance detected by qPCR reflects the localized reductions near where *Eimeria* has invaded the tissue. Deplancke and Gaskins (2001) suggested that acidic mucins have stronger defensive barrier functions than neutral mucins, preventing sporozoite access to the epithelial cell. Therefore, the decrease in Muc2 mRNA signals may be the result of the parasite manipulating expression of defensive genes of the intestine, likely by a similarly unidentified mechanism as the downregulation of LEAP2 discussed above.

## CONCLUSIONS

Following an *E. acervulina* challenge, infected chickens showed elongated crypts, and decreased LEAP2 and Muc2 mRNA starting at 3 dpi. During peak infection (5 and 7 dpi), VH and the mRNA abundance of LEAP2, AvBD1, AvBD6, AvBD10, and Muc2 were decreased in infected chickens compared to uninfected chickens. After peak infection VH of infected chickens rapidly increased compared to uninfected chickens to be equivalent at 14 dpi. The increase in VH was likely supported by increases in Col3a1 and Notch1 mRNA on 7 and 10 dpi; while the crypt elongation was reinforced by increased Ki67 mRNA following infection. A specific marker for an *E. acervulina* surface antigen gene was used to localize the site of *Eimeria* invasion in the duodenum. The Ea-SAG probe was only detected at 5 and 7 dpi. There was a pattern of suppressed Muc2 mRNA in regions of the duodenum where the Ea-SAG signal was present. Taken together these results provide a temporal analysis of host defense and cellular homeostasis genes during and after an *E. acervulina* infection that suggests a cause-and-effect relationship. At the cellular level, *Eimeria* manipulates host functions (defensive barrier genes) through an as of yet unidentified means likely to propagate the infection. In response, the intestine prioritizes regenerating villi (upregulation of homeostasis genes) resulting in crypt elongation and villi height recovery.
